# Editorial: Cognitive Reserve and Language Experience: Can Long-Term Use of Multiple Languages Protect Our Brains From the Effects of Aging?

**DOI:** 10.3389/fpsyg.2021.748181

**Published:** 2021-08-31

**Authors:** Roberto Filippi, Peter Bright

**Affiliations:** ^1^Department of Psychology and Human Development, Institute of Education, University College London, London, United Kingdom; ^2^School of Psychology and Sport Science, Anglia Ruskin University, Cambridge, United Kingdom

**Keywords:** bilingualism, cognitive reserve, bilingual advantage hypothesis, dementia—Alzheimer's disease, multilingualism

When we accepted the Frontiers in Psychology's invitation to take care of a second research collection after our previous successful “Perspectives on the ‘Bilingual Advantage': Challenges and Opportunities,” we could not predict the extremely challenging times ahead. Primary research, particularly when undertaken with human participants, has been difficult for everybody. We are therefore particularly thankful to the authors of the four excellent articles (one primary research paper and three reviews) included in this collection.

Ferreira et al. present a review of relevant work undertaken with professional interpreters in an attempt to resolve the ongoing debate on whether or not there is a genuine general cognitive advantage associated with (and underpinned by) the process of becoming bi/multilingual. These authors raise the issue of inconsistency in sample characteristics across studies, and, in particular, the wide heterogeneity in linguistic characteristics of those characterized as bilingual in this field. Their review outlines the unique bilingual experiences of interpreters, which demands real-time, high level auditory processing of one language while simultaneously translating and converting this input into verbal production in a different language. Although firm conclusions are not yet possible, due to the limited number of studies published to date, small samples, inconsistency in the tests employed and other issues, evidence is emerging for robust, short-term cognitive effects associated with intensive training in interpreting which may also underpin longer-term neurocognitive change.

In their ERP study of recovery from stroke-related aphasia, De Letter et al. explore pre-attentive (mismatch negativity, MMN) and attentive (P300) phonological discrimination ability in the chronic phase of post-stroke aphasia recovery across monolingual and bilingual groups. Across two time-points, they present evidence for a disproportionate improvement in phonological input processing speed in the bilinguals, indicated by a shortening of the MMN over time. While acknowledging that further research with larger groups is required, the authors consider the implications of their findings for diagnostics and rehabilitation in aphasic patients, and cognitive training in healthy aging people.

Teubner-Rhodes considers overlap in the neural networks underpinning multilingual language control and cognitive persistence, and presents a theory that multilanguage acquisition supports application of effort in cognitively demanding tasks in the service of better performance. In her review, the author identifies the anterior cingulate cortex and inferior frontal gyrus as the core network underpinning cognitive persistence. She presents evidence for different weighting of activity in these two regions across monolingual and bilingual groups during the execution of demanding tasks, consistent with more efficient cognitive control in bilinguals. She also raises implications of these group differences for the mitigation of age-related cognitive difficulties.

Heredia et al. provide an important critical review of the literature on bilingualism as contributor to cognitive reserve, and moderator of age-related neurocognitive deterioration. In contrast to inconclusive and counter arguments in recently published primary data articles and meta-analyses on the bilingual cognitive advantage, the authors interpret the literature to support bilingualism as both predictor and moderator of age-related changes in cognitive reserve. To help resolve the ongoing and fierce debate surrounding causal factors underpinning reported bilingual/monolingual group differences, the authors advocate new analytic approaches and the testing of far larger datasets than are typical in this literature, made possible through the availability of open and freely available repositories.

## Author Contributions

Both authors listed have made a substantial, direct and intellectual contribution to the work, and approved it for publication.

## Conflict of Interest

The authors declare that the research was conducted in the absence of any commercial or financial relationships that could be construed as a potential conflict of interest.

## Publisher's Note

All claims expressed in this article are solely those of the authors and do not necessarily represent those of their affiliated organizations, or those of the publisher, the editors and the reviewers. Any product that may be evaluated in this article, or claim that may be made by its manufacturer, is not guaranteed or endorsed by the publisher.

